# Structural analysis of the coronavirus main protease for the design of pan-variant inhibitors

**DOI:** 10.1038/s41598-023-34305-6

**Published:** 2023-04-29

**Authors:** Runchana Rungruangmaitree, Sakao Phoochaijaroen, Aunlika Chimprasit, Patchreenart Saparpakorn, Kusol Pootanakit, Duangrudee Tanramluk

**Affiliations:** 1 Institute of Molecular Biosciences, Mahidol University, Salaya, Nakhon Pathom 73170 Thailand; 2 Integrative Computational BioScience (ICBS) Center, Mahidol University, Salaya, Nakhon Pathom 73170 Thailand; 3 Department of Chemistry, Faculty of Science, Kasetsart University, Chatuchak, Bangkok 10900 Thailand

**Keywords:** Computational biology and bioinformatics, Drug discovery, Structural biology

## Abstract

With the rapid rate of SARS-CoV-2 Main protease (M^pro^) structures deposition, a computational method that can combine all the useful structural features becomes crucial. This research focuses on the frequently occurring atoms and residues to find a generalized strategy for inhibitor design given a large amount of protein complexes from SARS-CoV in contrast to SARS-CoV-2 M^pro^. By superposing large numbers of the ligands onto the protein template and grid box, we can analyse which part of the structure is conserved from position-specific interaction for both data sets for the development of pan-M^pro^ antiviral design. The difference in conserved recognition sites from the crystal structures can be used to determine specificity determining residues for designing selective drugs. We can display pictures of the imaginary shape of the ligand by unionising all atoms from the ligand. We also pinpoint the most probable atom adjustments to imitate the frequently found densities from the ligand atoms statistics. With molecular docking, Molecular Dynamics simulation, and MM-PBSA methods, a carbonyl replacement at the nitrile warhead (N5) of Paxlovid’s Nirmatrelvir (PF-07321332) was suggested. By gaining insights into the selectivity and promiscuity regions for proteins and ligands, crucial residues are highlighted, and the antiviral design strategies are proposed.

## Introduction

The unprecedented pandemic COVID-19 is still spreading globally with rising statistics of infection and mortality^1^. COVID-19 is caused by severe acute respiratory syndrome coronavirus 2 (SARS-CoV-2). One of the possible protein targets for the Coronavirus is the main protease (M^pro^). The main protease of SARS-CoV-2 plays an important role in processing the viral polyproteins to be the end products of the viral RNA for the cleavage site of the 1ab polyprotein, which is the replicase of the size 790 kDa^2^. After proteolytic cleavage by M^pro^, nsps (nonstructural proteins) are involved in the assembly of the viral replication transcription complex for the synthesis of the viral RNA^3,4^. M^pro^ plays an essential role in the encoding process which results in the formation of 2 polyproteins (pp1b and pp1ab). M^pro^ is then subsequently excised via self-cleavage. It also plays a crucial role in the cleavage of 11 other sites, thereby leading to the synthesis of 16 nonstructural proteins^5^. The coronavirus’s main protease, also known as M^pro^ or 3CL^pro^, is pivotal for the viral gene expression and replication. Its alternate name, 3C-like protease, signifies its structural resemblance to the 3C proteases found in picornaviruses as well as their shared substrate preferences^6^. There are 3 domains of M^pro^ in SARS-CoV-2 harbouring 306 amino acids, which are domain I (residues 8–101) with 4 α-helices and 7 β-strands, domain II (residues 102–184) with 7 β-strands, and domain III (residues 201–303) with 5 α-helices^7–9^. M^pro^ is required to be dimerised from protomers A and B in order to be activated. This process is facilitated by the flexibility of domain III^9^. Several studies have shown that the main protease of SARS-CoV and SARS-CoV2 is conserved in terms of both sequence and structure. M^pro^ of SARS-CoV-2 is closely related to other severe acute respiratory syndrome coronaviruses (SARS-CoV) as it serves an equivalent function^2^. The inhibition of this enzyme would potentially stop the viral protein from replicating in the host system. In addition, it should be harmless to humans because there is no known SARS-CoV-2 M^pro^ cleavage site in humans^2^. Therefore, M^pro^ of SARS-CoV-2 is widely used as a target for drug design.

Different variants of concern, such as Delta (B.1.617.2) and Omicron (B.1.1.529), lead to an onset of long-term effects of COVID-19^10^. Recently, there are very promising interferon treatment that is shown to cure multiple variants after the patients have been infected with COVID-19^11^. However, they are delivered via subcutaneous injection and also require special storage. The way to modify orally administered drug in the form of small molecule, such as Nirmatrelvir of Paxlovid, to prepare antiviral pills for new variants in the near future is therefore crucial. Thus, a computational approach that can aggregate the structural information, and able to divide the structural features into promiscuous parts and selectivity parts for small molecule inhibitor design is required. This research aims to provide alternative methods for drug design, which might be helpful to analyse pan-M^pro^ variant inhibitors. Here, our computational approach provides insights into SARS-CoV-2 M^pro^ inhibitor design that can be quickly done with low computing power. The process can be easily applied to other classes of protein inhibitors. The approach implemented in this study is based on the software called SIMFONEE (Specificity IMplication from Frequently Occurring NEighboring Entities).

Even though, molecular docking has been introduced to help screening for drug compounds against M^pro^ SARS-CoV-2^12^, the energy estimation and docking pose alone is far from accurate^13^. A very promising way is to tailor the ligand molecules based on various binding site prediction tools^14^. To enhance the binding site analysis, our study introduces an extra step to analyse pockets of the aggregated dataset of M^pro^ SARS-CoV-2 onto a template via SIMFONEE package. We provided a way to capture atomic locations of the conserved atoms by intensifying the signal-to-noise ratio of the neighbouring atoms and supported the results with MD simulations.

The idea to derive the position specific interaction is originated from the image processing of Electron Microscopy which superposes large amounts of micrographs to increase the signal from noise in the background^15^. To make an analogy, protein structure deposited in the protein databank contains thousands of crystal structure of amino acids together with their chemical partners at certain distance threshold. When we superposed pictures of these amino acids from high resolution PDB coordinates, the signals of the highly favourable interacting partners can be obtained^15^. We applied the same ideas to capture these frequently occurring atoms within the structure of M^pro^. In this way the signal to noise ratio of the binding partner atoms can be intensified. At the same time, the voluminous structure from union of ligand atoms can be used to probe ligand accessible locations from more than 300 M^pro^ structures. The provided result can estimate the shape of the imaginary ligands for the drug candidates so that downstream chemical modifications can be further elucidated. The experimental design for this study includes three main steps. Initially, the phylogenetic analysis of M^pro^ Coronaviruses in SARS-CoV and SARS-CoV-2 were done for both sequences and structures to prove that the two classes of coronaviral M^pro^ structures are closely related. The superposition of both SARS-CoV and SARS-CoV-2 M^pro^ is used to determine the frequently occurring atoms of M^pro^ from various Coronavirus species. Although, these frequently occurring atoms may not interact directly with the drug molecule, our previous studies showed that they can be used as anchoring points for measuring the influential distances that can guide ligand design^16,17^. Then, the structures of M^pro^ SARS-CoV-2 are analysed to show the parts that are position-specific via our 4-dimensional grid analysis. Lastly, the investigation of the active site boundary is compared with the binding energies from derivatives of existing drug component from Paxlovid (PF-07321332 or Nirmatrelvir), which is the M^pro^ inhibitor from Pfizer^18^. Via these steps, drug design for the future emerging diseases with sufficient targeting crystal structures can be facilitated using SIMFONEE.

## Materials and methods

### Phylogenetic tree analysis to show relationship among M^pro^ Coronavirus sequences

Seventeen nonredundant M^pro^ sequences of Coronaviruses, which include SARS-CoV, SARS-CoV-2, MERS-CoV, human coronavirus NL63, human coronavirus HKU1 (isolate N1), Tylonycteris bat coronavirus HKU4, Porcine epidemic diarrhea virus, Porcine transmissible gastroenteritis virus, Feline infectious peritonitis virus, Murine hepatitis virus strain A59, Betacoronavirus England 1, infectious bronchitis virus, and Cavally virus, were selected. High resolution structures and sequences were downloaded from the RCSB Protein Data Bank. The molecular mass and net charge of each entry were computed from Biovia’s Discovery Studio^19^. The sequence and structure relationship among the family and the percent identity of each coronavirus M^pro^ were obtained from NCBI’s Blast^20^. The phylogenetic tree was reconstructed by MEGA11^21^. The calculated mass and charge were visualized as heatmap using iTOL^22^.

### Dataset preparation

Two separate datasets of SARS-CoV and SARS-CoV-2 were prepared^23^ and selected by using the PISCES web server based on resolution of the structures^24^. The data were collected up until 27 December 2021. The data set comprises 102 structures of SARS-CoV and 397 structures of SARS-CoV-2 M^pro^ (Supplementary 1). The structure with PDB ID: 6XHN was used as the M^pro^ superposition template for SARS-CoV and PDB ID: 7K3T was used as the template for SARS-CoV-2 using SIMFONEE package. The results were visualised based on various cut-offs of the frequency found in a 4-dimensional grid^15^. The result was visualised via PyMOL^25^.

### Atomic level analysis for inhibitor design

**Figure 1:**
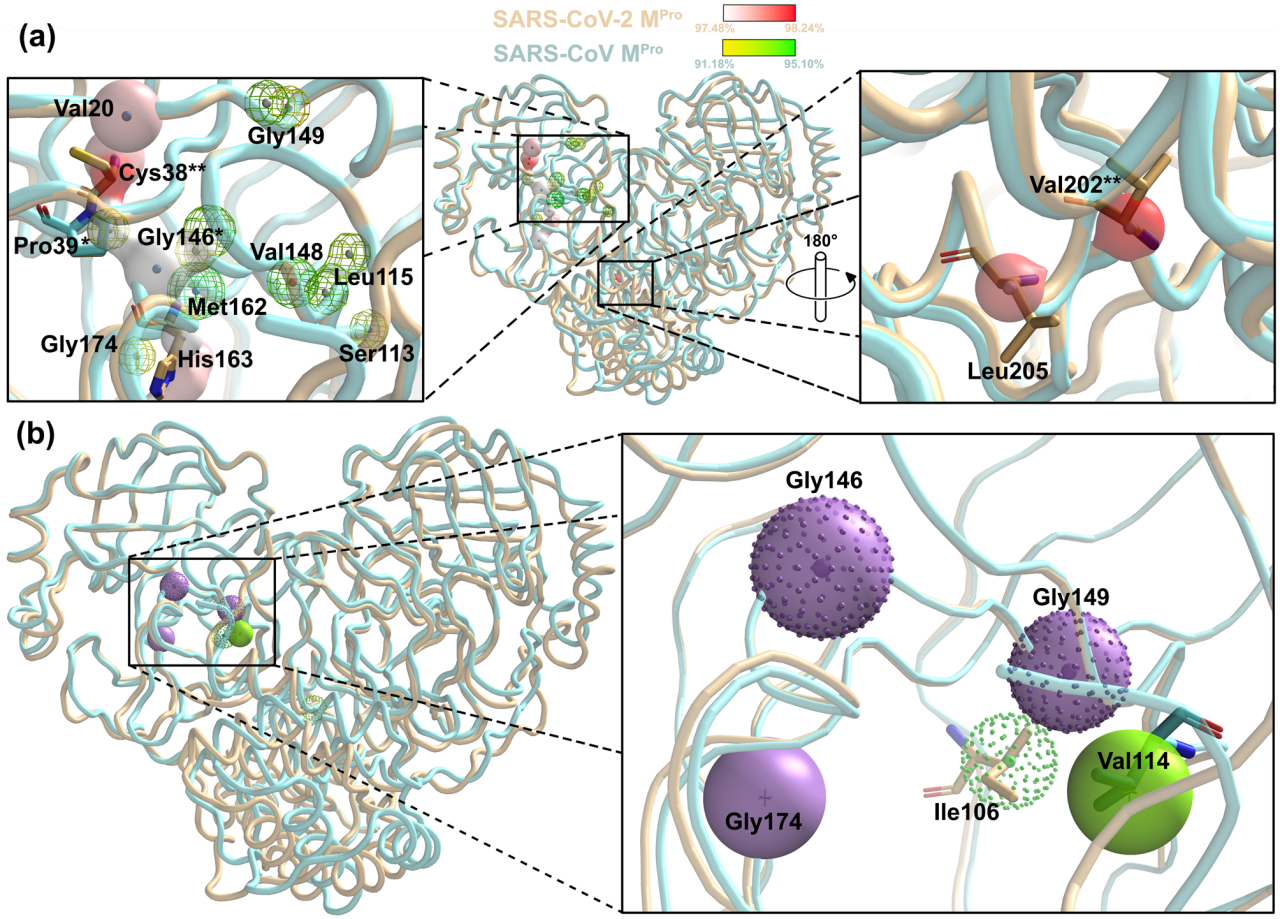
Selectivity and promiscuity of SARS-CoV-2 M^pro^. (**a**) The frequently occurring atoms of the residues of SARS-CoV (yellow-green gradient mesh representation) and SARS-CoV-2 (white-red gradient surface representation) M^pro^ (SARS-CoV M^pro^: aquamarine cartoon tube, SARS-CoV-2 M^pro^: beige cartoon tube) with small spheres indicating conserved atoms (CPK colour). The most conserved part for SARS-CoV-2 is at Cys38 while the ones for SARS-CoV is shifted down at the location surrounding the mainchain oxygen of Val148. (**b**) The superposed M^pro^ structures of SARS-CoV and SARS-CoV-2 illustrating the key conserved residues among each dataset (transparent spheres: frequently occurring residues from SARS-CoV; dot clusters: frequently occurring residues from SARS-CoV-2). Gly146 and 149 are the anchoring residues presented from both data sets (purple sphere and overlapped with dots). These main chain atoms of glycines can be the location for pan-coronaviral drug design. The Ile106 and Ala206 (green dots) are unique position specific residues in SARS-CoV-2 data set and thus can be specificity determining residues. The figure was created by using PyMOL Version 2.5.3 (https://pymol.org)^25^.

SIMFONEE package was implemented to pinpoint the most frequently occurring atoms of the residue from both SARS-CoV and SARS-CoV-2 M^pro^, where we wanted to find the overlapping of the 2 data sets and infer that they are the pan-variant recognition site. We wanted to find position-specific atoms based on the repeating occurrence on a 4-dimensional grid, which we determined from superposition and called them the conserved atoms. These conserved atoms had the same Amber atom types^26^ such as SP2 and SP3 carbon atoms, and we counted their occupancies from the high resolution X-ray crystal structures. For the residues that are position specific, we determined the recurrence of atoms that have the same types and rounded numbers of Cartesian coordinates by collecting penultimate atoms at the distinctive part of the residues in 3D structures^17^. The algorithm of the software aims to increase the signal-to-noise ratio from summing up the counts from atomic superposition. The 4-dimensional grid was used to collect the atom occupancies from the superposed structures from the data set and yields the [x][y][z] coordinates along with the types of atoms and residues. These count numbers were collected in a 4D-array. The most populated atoms were visualized as spheres with colours according to atom types on PyMOL^25^. Various cutoff numbers were used to observe the distinct position-specific interactions. These positions-specific entities were filtered from more than 300 crystal structures of SARS-CoV-2 and only those which were at > 97% conservation were used. The software can also provide the overlay of all the ligand accessible space and shown differently as wire mesh for the SARS-CoV M^pro^ dataset and as surface representation for SARS-CoV-2 M^pro^ (Fig. 1a). The retained residues are also represented as dots (SARS-CoV-2) and sphere (SARS-CoV) with colours according to the amino acid side chains (Fig. 1b).

### Mapping of the active site protomer A of M^pro^ SARS-CoV-2

The graphical blueprint of the bound ligand mapped to the target is predicted by the software SIMFONEE and verified by drugs which passed phase 3 of the study. Data preparation and selection are similar to the conserved atoms identification procedure. All the atoms that were harvested from the 4-dimensional grid were summed up. Positions that were observed more frequently than random fluctuation (> 2%) were pooled and displayed on the same orientation as the M^pro^ template. The oxygen atoms of water and sulphur atoms from the crystallising reagents were omitted from the results, leaving only the main atom types of nitrogen, oxygen, and carbon to be presented as wire mesh representation for the imaginary ligands in the vicinity of the M^pro^ active site on PyMOL. This method was made to include the highest possibility of the drug ligand’s atom type. They were presented as small spheres which are coloured according to the element type (CPK colours).

### Molecular docking studies

Crystal structure of SARS-CoV-2 main protease in complex with protease inhibitor from Paxlovid, which are PF-07321332 or also called Nirmatrelvir, (PDB ID: 7vh8) was taken from the RCSB Protein Databank^27^. We focus on PF-07321332, its carbonyl derivative (compound 1), and its hydroxyl derivative (compound 2). Covalent docking was applied in order to investigate how these compounds bound via covalent bond to Cys145. Covalent constraint was set between a sulfur atom of Cys145 and a modified carbon atom of cyano group. In order to investigate binding modes of PF-07321332 and proposed compounds, a covalent bond between a sulfur atom of Cys145 and a carbon atom of cyano group in PF-07321332 was deleted. The orientation of cyano group was adjusted. The proposed compounds were constructed based on the PF-07321332 structure by replacing the cyano group with carbonyl (compound 1) and hydroxyl group (compound 2), respectively. Molecular docking using GOLD2022.2.0^28^ was applied to examine the binding of PF-07321332 and proposed compounds into the SARS-CoV-2 main protease. The validation of docking parameters was tested. The binding area was defined based on the location of PF-07321332 and atoms within 12 Å away from PF-07321332. The default parameters were set and a genetic algorithm (GA) run of 100 were used. Goldscore scoring function was used and the docked conformations with the highest Goldscore were selected.

### Molecular dynamics simulations

The selected docked conformations of PF-07321332 and compounds 1–2 were further performed the molecular dynamics (MD) simulations using GROMACS version 2022.1^29^. Dimer form was constructed by superimposition to another chain. The AMBER99SB protein force field parameter was chosen. The complexes were solvated using TIP3P water model about 10 Å from the surface in cubic box. MD simulations about 100 ns were performed. Root-mean-square deviation (RMSD) was investigated. Root-mean-square fluctuation (RMSF), the radius of gyration, the number of hydrogen bond and cluster analysis were studied using the MD trajectories during 90–100 ns. After that, the binding free energies (∆G_binding_) of the 1001 trajectories between 290 and 300 ns of the compounds was applied using molecular mechanics Poisson − Boltzmann surface area (MM-PBSA)^30^ in GROMACS. The structures were visualised by Biovia Discovery Studio^19^.

## Results and discussion

### Sequence and structure relationship among Coronaviruses M^pro^

M^pro^ of SARS-CoV (PDB ID: 6XHN) and SARS-CoV-2 (PDB ID: 7K3T) are significantly related as shown in the phylogenetic tree and the heatmap representation (Supplementary 2). This relationship among the different main protein sequences, or even among different variants of SARS-CoV-2 M^pro^, can be further used for pan-variant inhibitor design.

As expected, these results showed a close relationship between SARS-CoV and SARS-CoV-2 M^pro^. There is a high conservation between charge properties of the main proteases from SARS-CoV and SARS-CoV-2. The similarity in sequence, structure, and charge suggested that dual-specific drugs can be designed. The conserved atoms and residues between SARS-CoV and SARS-CoV-2 may be targeted and a general rule to target several variants via these promiscuous parts of the protein may be obtained.

### Position-specific residues enable the design of pan-SARS-CoV-2 M^pro^ inhibitor

To date, many variants of concern were reported by Outbreak.info, whereby the difference in amino acid sequences among SARS-CoV-variants are presented^31^. The approach to guide the design of inhibitor which can potentially bind to multiple variants of SARS-CoV-2 are needed. SIMFONEE can be used to identify position specific residues, which are the shared conserved amino acid positions from a 4-dimensional grid analysis of M^pro^ of SARS-CoV and SARS-CoV-2. For example, in this study, key conserved residues across Coronaviruses M^pro^ were found to be Pro39, Gly146, and Met162 (Table 1, asterisk). Met162 has been discovered to be conserved in SARS-CoV for its nitrogen atom at 95.10%, while being conserved in SARS-CoV-2 at 96.98% for its alpha carbon atom (Fig. 1a, Table 1). The delta carbon atom of Pro39 is conserved in 97.48% of SARS-CoV-2 versus 92.16% in SARS-CoV. While carbon (93% conservation) and oxygen atoms (95% conservation) of Gly146 are found out of 102 SARS-CoV M^pro^ structures, carbon and nitrogen atoms of Gly146 are frequently occurring at 97.48% (387 structures from 397) in SARS-CoV-2 M^pro^. Moreover, Pro39 and Gly146 were found to be in close proximity. Furthermore, Pro39 mutation was reported to lead to M^pro^ instability^32^. This Pro39 was also reported to interact with 2,4-dichloro-5-methylbenzene in a hydrophobic groove of M^pro^ SARS-CoV^33^. Hence, this motif could be a target for the design of a pan-Coronavirus M^pro^ inhibitor. Five amino acids, Val20 (nitrogen atom: 97.73%), Cys38 (alpha carbon and nitrogen atoms: 98.24% and 97.73%), His163 (beta carbon: 97.48%), Val202 (alpha carbon: 98.24%) and Leu205 (beta carbon: 97.98%), are predicted to be very specific to SARS-CoV-2 (Fig. 1a). Inversely, the SARS-CoV M^pro^ position specific conservation is shown as an alpha carbon atom of Ser113 (92.16%), carbon and a beta carbon atom of Leu115 (93.14% and 92.16%), and oxygen atom of Val148 (94.12%) (Fig. 1a). Therefore, the most conserved part for SARS-CoV-2 is at Cys38 and Val202 (Table 1, double asterisks), while the most conserved ones for SARS-CoV is the location surrounding the main chain atoms of Val148.

**Table 1:** The comparison of the atom types and percentage of atom conservation.

Amino acid	Residue	SARS-CoV			SARS-CoV-2			Reported literature
		%	Atom type	Score	%	Atom type	Score	
Cys	16	91.18	C	93				
Met	17	93.14	C	95				
Val	20				97.73	N	388	
Gly	29				96.98	N3 (N)	385	
Cys**	38				98.24	CT (CA)	390	
					97.73	N	388	
Pro*	39	92.16	CT (CD)	94	97.48	CT (CD)	387	Bzówka et al.^32^
								Lu et al.^33^
Ser	113	92.16	CT (CA)	94				
Leu	115	93.14	C	95				
		92.16	CT (CB)	94				
Gly*	146	91.18	C	93	97.48	C	387	
		93.14	O	95	97.48	N3 (N)	387	
Val	148	94.12	O	96				
Tyr	161				97.73	C	388	
					97.73	OH	388	
Met*	162	95.10	N	97	96.98	CT (CA)	385	
His	163				97.48	CT (CB)	387	Owen et al.^18^
								Rossetti et al.^40^
His	172				97.23	CT (CA)	386	
Ala	173				97.48	N	387	
Gly	174	91.18	CT (CA)	93				
Leu	177				97.23	O	386	
Val**	202				98.24	CT (CA)	390	Günther et al.^36^
								Kumar et al.^39^
								Sultan et al.^38^
								Verma et al.^37^
Leu	205				97.98	CT (CB)	389	

The data for each amino acid residue position from SARS-CoV versus SARS-CoV-2 M^pro^ with modified AMBER atom types are compared.

*CT* any sp3 carbon, *CA* C-alpha carbon, *CB* C-beta carbon, *CD* C-delta carbon of the amino acids, for further explanation please refer to Supplementary 1 and 2 of Tanramluk^17^.

*The pan-Coronaviral specific residues between SARS-CoV and SARS-CoV-2 M^pro^*.*

**The most conserved residue in SARS-CoV-2 M^pro^*.*

For side chain comparison of the position specific residue sidechains, Ile106 and Ala206 are SARS-CoV-2-specific; whereas, Val114 and Gly174 are only specific to SARS-CoV M^pro^ (Fig. 1b). In contrast, Gly146 and Gly149 can be seen to be retained in both SARS-CoV and SARS-CoV-2 M^pro^ as shown with overlapped sphere with dot representation (Fig. 1b).

### Cys38 and Val202, the most conserved residues of SARS-CoV-2 M^pro^

For SARS-CoV-2 M^pro^, the alpha carbons of Cys38 and Val202 showed a very high conservation for position and type, i.e. 390 out of 397 M^pro^ structures or 98.24% (Fig. 1a). Although Cys38 is reported to be resided in the hydrophobic cavity of SARS-CoV-2 M^pro^ and protected from external interaction by the negative charges according to Kneller and colleagues in 2020, it is explored in our study that Cys38 is one of the most conserved M^pro^ SARS-CoV-2 residues^34^. On the other hand, Val202 is located in the vicinity between domains II and III of M^pro^ SARS-CoV-2 which reportedly may be the binding sites for Sumarin and other compounds^6^. This valine may lead to the diminished flexibility of domain III as well as disrupting the M^pro^ structural dimerization^8^. The interaction between the compounds and the residues in domain III is also crucial for the design of a drug that would tightly fit to the target. Several studies have revealed that domain III of SARS-CoV-2 (residues 201–303), in which Val202 belongs to, plays a major role in dimerisation of the protein structure through the interaction between 5 alpha helical domains. Furthermore, the dimerisation of SARS-CoV-2 M^pro^ is necessary for enzyme activation as its oxyanion loop is required to be stabilised for catalytic activity^9,35^. It was suggested that Val202 may be associated with the binding of AT7519 by the van der Waals interaction to the dichlorobenzene ring of the compound in the allosteric site where it binds to the depth of the cavern in the middle of the dimerisation and catalytic domain^36^. Val202 also forms Pi-alkyl bonds to several compounds from different studies, i.e. canthin-6-one 9-0-beta-glucopyranoside and Neoandrographolide^37^, SRT2183 and SRT1720^38^, and Sesamolinol^39^. These studies confirmed our findings in which Val202 is discovered to be one of the most highly conserved residue of SARS-CoV-2 M^pro^ since it serves an important function in viral enzyme activation.

His163 was also predicted to be crucial. The alpha-carbon atom of His163 showed high position-specific conservation for SARS-CoV-2 at 97.48% and hence may be used when considering species selectivity instead of pan-M^pro^ inhibition for the promiscuous ones. The side chains of His163 (3.1 Å) and His172 (2.9 Å) were studied extensively in 2022^40^. A dihydro‑quinolinone compound, Z222979552, showed an inhibition via hydrogen bond interactions between the tested compound and these two histidine residues. His163 was also reported to interact between the binding of SARS-CoV-2 M^pro^ and Paxlovid (PF-07321332)^18,41^. Cys145 and His 41, which are keys to the mechanisms^27^, are not found to be position specific.

As can be seen from the rediscovery of these aforementioned residues, SIMFONEE can be used to confirm or discover crucial binding atoms computationally. Importantly, it can be used to predict the anchoring points for drug inhibitor design according to our previous work^16^.

### Imaginary shape of the ligand

**Figure 2:**
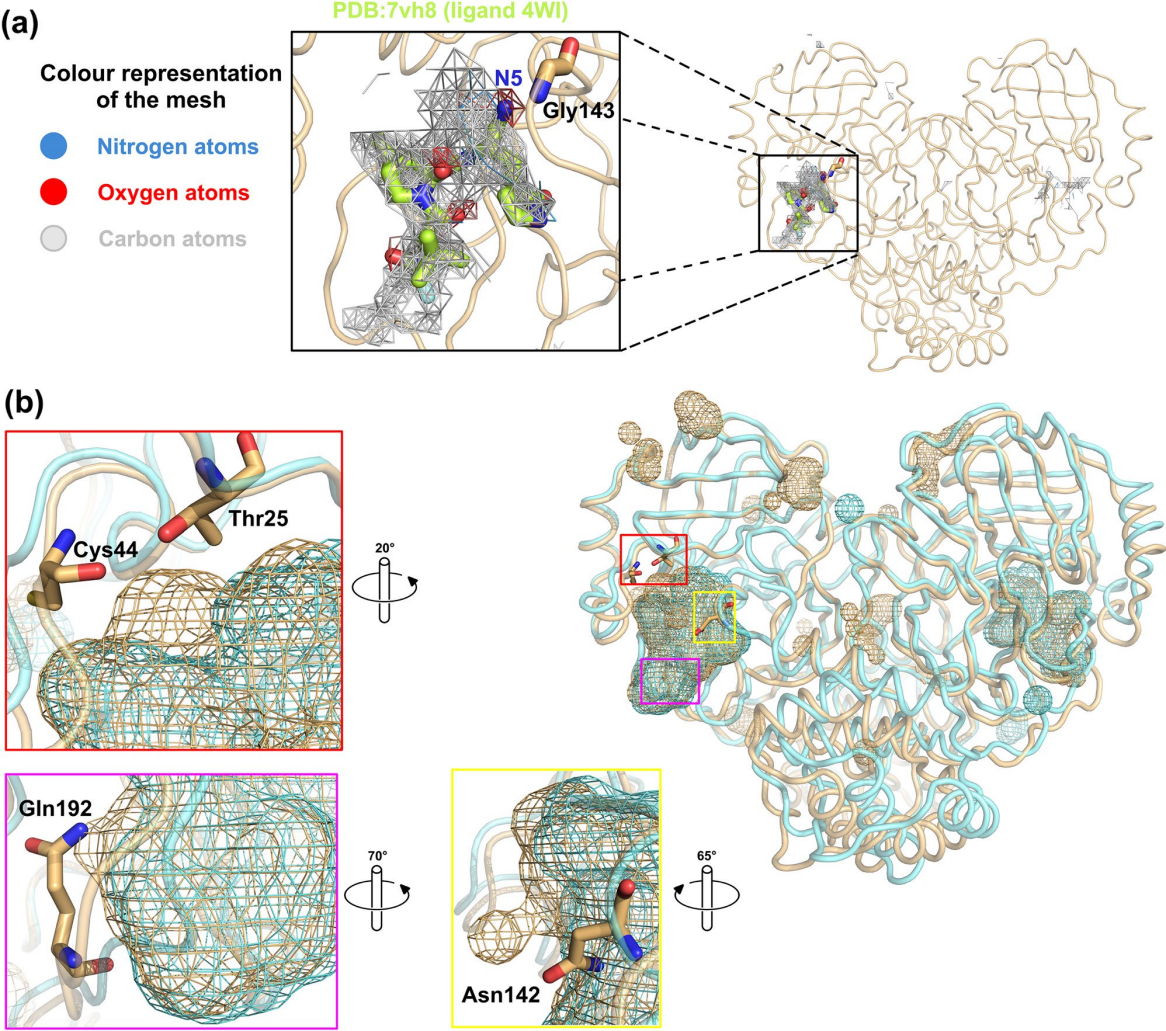
Imaginary shape of M^pro^ binding pocket. (**a**) The graphical blueprint of the active site boundary of imaginary ligands to SARS-CoV-2 M^pro^. The mapping in mesh representation shows nitrogen atoms as blue mesh, oxygen atoms as red mesh, and carbon atoms as grey mesh. Paxlovid’s Nirmatrelvir (PF-07321332) with ligand’s code: 4WI (PDB:7vh8 in protomer A) is in stick representation with its carbon atoms in lemon colour. The mesh on the left-hand panel shows nitrogen at position N5 (blue), from 4WI exists at the location where the majority of the data from large datasets suggested that this location should be oxygen (red mesh harbouring N5 of 4WI, top right). This oxygen can interact with nitrogen from the main chain of Gly143. Nirmatrelvir (PF-07321332) occupies most of the space but there are still areas that more atoms can be added (gray mesh represents carbon atom location from the ligands which are found surrounding PF-07321332, lemon sticks). (**b**) The interacting residues, i.e. Thr25 and Cys44 (highlighted in red, rotated 20°), Asn142 (highlighted in yellow, rotated 65°), and Gln192 (highlighted in magenta, rotated 70°), locates in a close proximity to the SARS-CoV-2 M^pro^-specific ligand atoms of the active site boundary (beige mesh blobs). Cyan mesh blob identifies the binding boundary of SARS-CoV M^pro^. The figure was created by using PyMOL Version 2.5.3 (https://pymol.org)^25^.

SIMFONEE can predict the shape of an imaginary ligand, which would bind to the pocket of the protein as confirmed by several drugs such as Paxlovid^15,41,42^. The graphical blueprint of the active site boundary of SARS-CoV-2 M^pro^ (beige cartoon tube representation) reveals the predicted active site boundaries collected from the non-random ligand atoms of the deposited SARS-CoV-2 M^pro^ PDB structures (Fig. 2a). Non-random atoms refer to the atoms of the ligands which occurred in the same grid box more than 2% within the input protein structures according to the SIMFONEE algorithm. The mesh obtained by the unionising all the non-random ligand atoms portrayed a merged map of > 300 ligands from SARS-CoV-2 M^pro^ X-ray crystal structures that were deposited in the PDB. The output is in the form of a voluminous structure with the shape similar to Paxlovid’s PF-07321332 (PDB-3-letter code ID: 4WI from PDB code: 7VH8). The chemical structure of PF-07321332 is not included in the input dataset but the density occurred to be similar to the voluminous structure obtained from superposition of unrelated ligand atoms. The imaginary shape of the ligand in the pocket of M^pro^ is found located in the vicinity of domain II and III (Fig. 2a); whereby, Paxlovid’s Nirmatrelvir (PF-07321332) fits well in this pocket. However, there are some results which are not according to the predicted map. For instance, the nitrogen atoms were not predicted for most parts of the ligand 4WI (Fig. 2a). However, all oxygen atom orientations of Nirmatrelvir fit right in the map according to the prediction. As a result, we reported that the methods can be used to construct an imaginary shape of the drug based on collection of all existing ligand atoms from input structures. We propose the possible atoms to be replaced on the nitrogen (N5) on the cyano group of Nirmatrelvir (Fig. 2a) and some further modification to be added in the empty vicinity from the map as a novel designed part of the drug molecule (Fig. 2b).

For a comparison of the fitted binding pocket, PDB ID: 7vh8 (ligand code: 4WI), which is the compound PF-07321332 (Nirmatrelvir) of Paxlovid, is illustrated. From protomer A of M^pro^, a nitrogen atom, N5, of the ligand 4WI is not according to the map, which recommends that the majority of ligands have oxygen at the location as indicated in the red colour mesh (Fig. 2a). This investigation might suggest that the drug that occupies the majority of the space in the mapping is more likely to be efficient at viral inhibition since it has more interaction to neighbouring residues of the M^pro^ structure. There are several atoms of the ligand which interact with SARS-CoV-2. These specific ligand atoms are shown as blobs protruding out from the overlay pictures of the mesh between SARS-CoV and SARS-CoV-2 active site boundary (protomer A). Thr25, Cys44, Asn142, and Gln192 are the interacting residues to the atoms of the ligand boundary, which are specific to SARS-CoV-2 M^pro^. Most of the ligand atoms of SARS-CoV are also in the same vicinity as SARS-CoV-2 and hence found to be overlapped (Fig. 2b).

### Molecular docking

**Figure 3:**
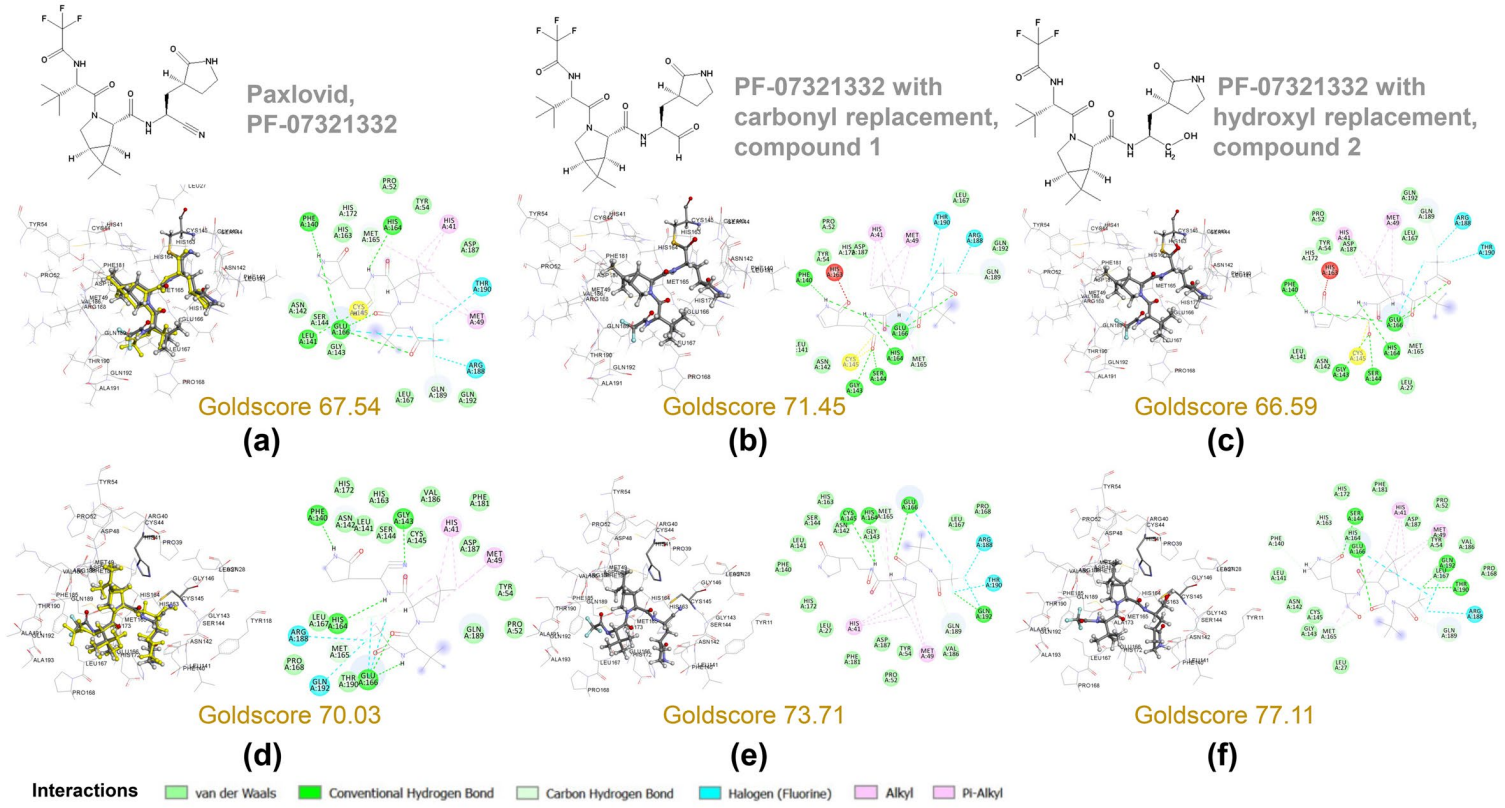
Covalent docking (**a–c**) and non-covalent docking (**d–f**) studies of Nirmatrelvir (PF-07321332) derivatives. Docked orientation of PF-07321332 (**a,d**) as compared with the crystal structures (yellow). Interactions to the residues in the binding pocket are shown. The carbonyl replacement at cyano group of PF-07321332 as supported by the 4D-grid analysis is shown by compound 1 (**b,e**). A hydroxyl replacement is not a good option from covalent docking studies, but it reveals a better fitness score via non-covalent binding as shown in compound 2 ((**c,f**), respectively). PF-07321332 with carbonyl replacement has better fitness scores than PF-07321332 for both covalent and non-covalent binding. The figure was created by using BIOVIA Discovery Studio Visualizer Software, Version 21.1.0.20298^19^.

From the covalent docking of PF-07321332 derivatives, the docked orientations of carbonyl replacement (compounds 1), and hydroxyl replacement (compound 2), were well oriented as compared with the PF-07321332, as shown in Fig. 3a–c. Their Goldscores were 67.54, 71.45, and 66.59 for PF-07321332, compound 1, and compound 2, respectively. The Goldscore suggested the possibility of carbonyl replacement to covalently bind to Cys145. The mechanism for the reaction of this carbonyl as an aldehyde group covalently bound with cysteine residue was previously described^43,44^. Their interactions to the amino acids in the binding pocket were also similar to the interactions found with PF-07321332. For the non-covalent binding of the PF-07321332 and compounds 1 and 2, molecular docking to the structure for initial binding was performed. The validation of the docking revealed the good reproducibility of PF-07321332 in the binding pocket with the RMSD of 0.718 Å, as shown in Fig. 3d. The Goldscore of PF-07321332 is 70.03. For the proposed compounds, their Goldscores revealed the better fitness score with the values of 73.71 and 77.11 for compounds 1 and 2, respectively. Therefore, the oxygen replacement from hydroxyl group from both covalent and non-covalent docking showed that this is not a good option (Fig. 3e,f). Their docked orientations and interaction to the residues in the binding pocket are shown in Fig. 3b,c. From the docking results, the orientation of compounds 1 and 2 were similar to the docked PF-07321332. Their interactions in the binding pocket were also similar. The interactions to His41 and Cys145 which are key interactions were still found. In case of the docked compound 1, the replacement with carbonyl group caused the stronger hydrogen bond interaction of NH group at the middle to Cys145. For the compound 2, the additional interaction from the 2-pyrrolidone ring to the residues were found as follows; a carbonyl group to His163 and Ser144, and a CH_2_ group to Phe140 and Glu166. In order to determine the binding interaction of the compounds at equilibrium, MD simulations were then performed.

### Molecular dynamics simulations

**Figure 4:**
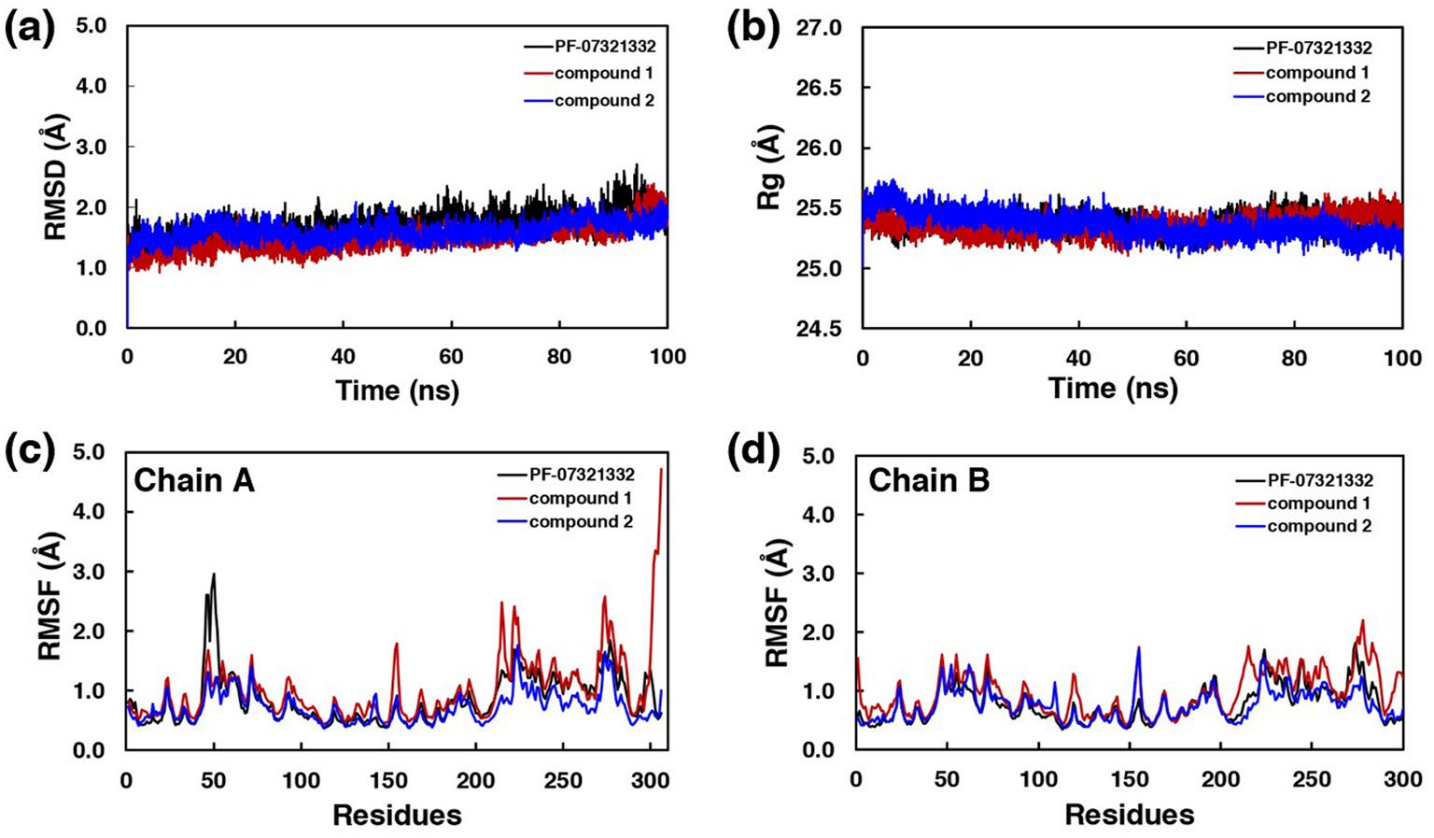
Molecular dynamics simulations. (**a**) Root-mean-square deviation (RMSD), (**b**) the radius of gyration, and root-mean-square fluctuation (RMSF) of (**c**) Chain A and (**d**) Chain B.

The MD results revealed the stability of the binding as represented in the RMSD results with the fluctuation less than 1 Å as shown in Fig. 4a. The radius of gyration showed the similar compactness in all systems, as shown in Fig. 4b. The fluctuations of the residues in both chains during 90–100 ns MD trajectories were also reported in Fig. 4c,d.

In chain A, the fluctuations of the loop (Ser46 to Asn51) close to the binding site of PF-07321332 and the loops (Gly215 to Asp216 and Arg222 to Thr224) of the compound 1’s complex were found. In case of chain B, low fluctuation was found throughout the structure.

**Figure 5:**
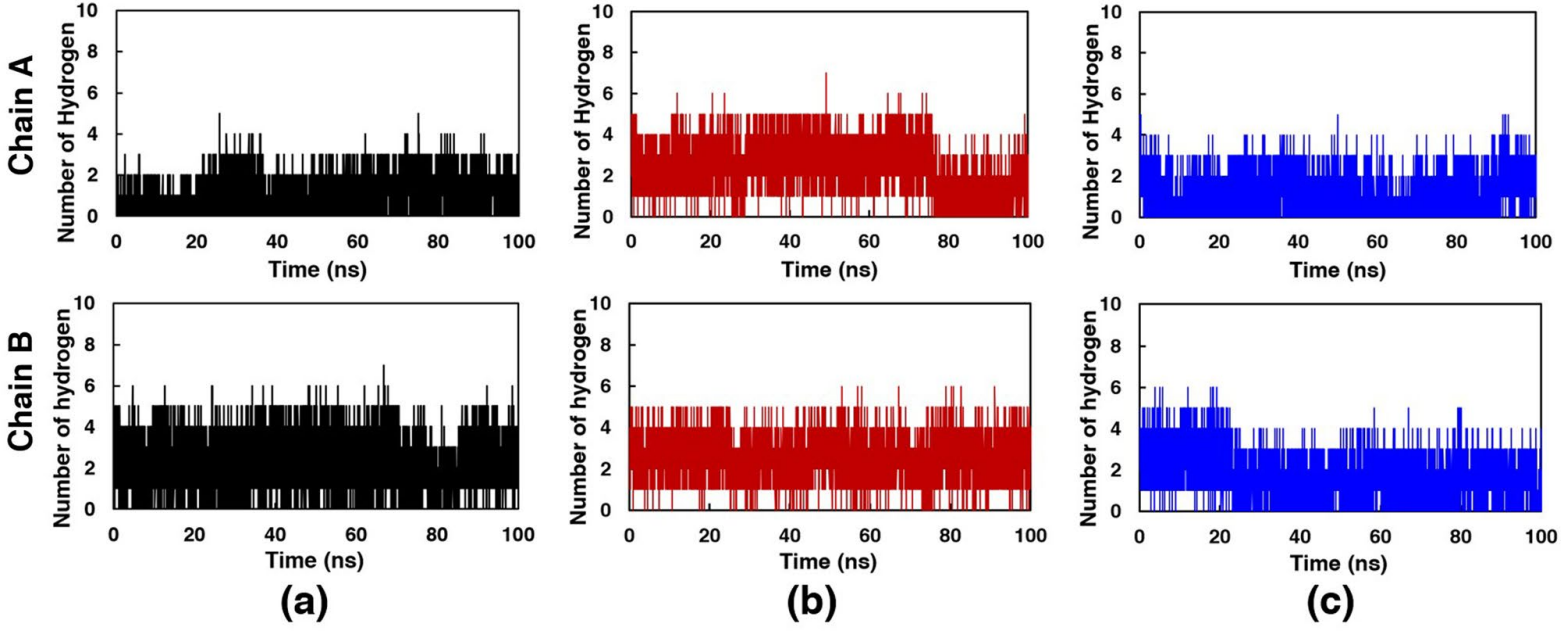
Number of hydrogen bonds. (**a**) PF-07321332, (**b**) compound 1 and (**c**) compound 2, with hydroxyl replacement, in both chains of SARS-CoV-2 main protease along the simulation time.

Hydrogen bond (H-bond) interaction between compounds and the structure of SARS-CoV-2 main protease was investigated as shown in Fig. 5.

All compounds revealed the H-bond with the residue in both chains throughout the simulation times. In both chains, compound 1 showed the higher average number of H-bond than PF-07321332 and, compound 1 respectively, during 90–100 ns. During 90–100 ns, the average numbers of H-bond were about 1.15, 2.49, and 0.98 in chain A and 2.28, 2.55, and 1.77 in chain B for PF-07321332, compound 1, and compound 2, respectively. The results indicated more average H-bonds in both chains of compounds 1 with SARS-CoV-2 main protease than that of PF-07321332. It may be assumed for better binding of compounds 1 than PF-07321332.

**Figure 6:**
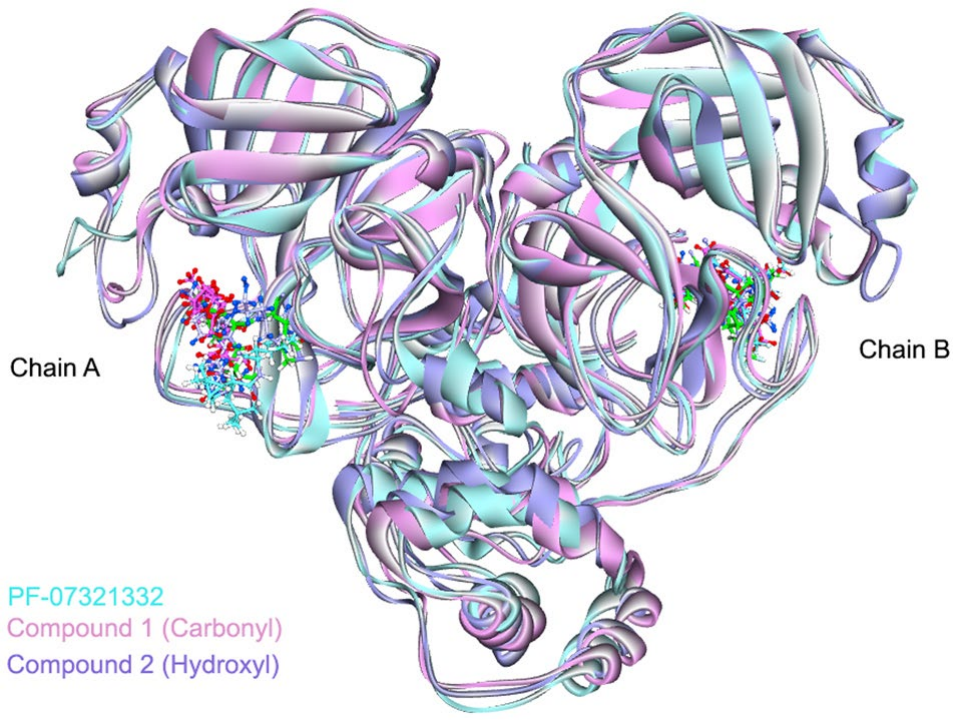
The superimposition of the complex with PF-07321332. The figures are shown using atom-type colour scheme, where the crystal structure of the ligand PF-07321332 from PDB ID:7VH8 is shown (green sticks). The structures with PF-07321332 (cyan), compound 1 (pink) and compound 2 (purple) are obtained from cluster analysis (90–100 ns). The figure was created by using BIOVIA Discovery Studio Visualizer Software, Version 21.1.0.20298^19^.

The superimposition of the structures from cluster analysis are shown in Fig. 6.

**Figure 7:**
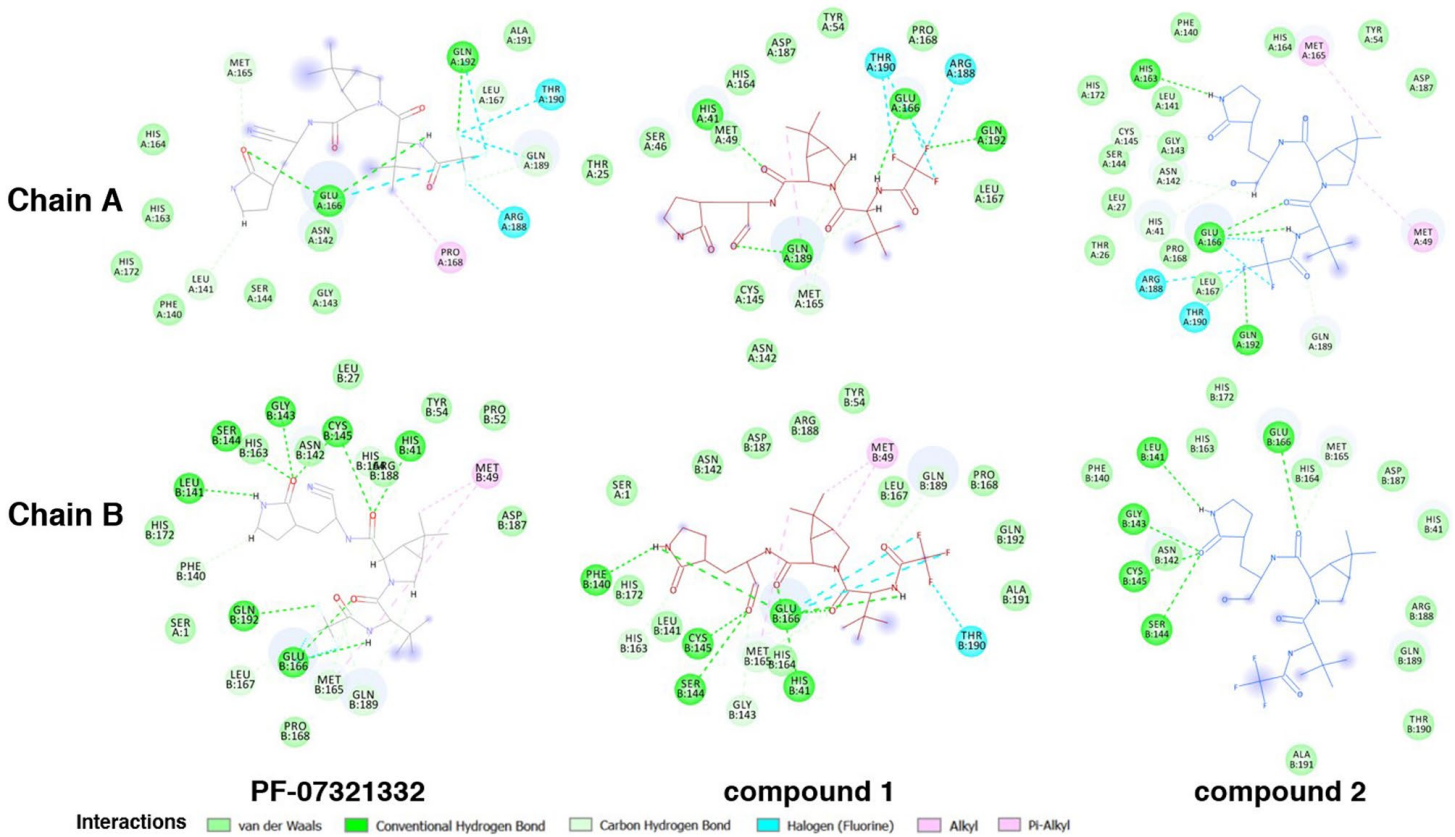
Interactions between the compounds and the residues in the binding pocket. The structures were obtained from cluster analysis at 90–100 ns. These figures to depict interacting partners from MD simulations were created by using BIOVIA Discovery Studio Visualizer Software, Version 21.1.0.20298^19^.

In chain A, due to the high fluctuation of the loop close to the binding site of PF-07321332, the location of PF-07321332 was slightly moved away from the binding site. For compound 1 in chain A, the location of 2-pyrrolidone ring was slightly different as compared to the crystal structure of PF-07321332. The location of compound 2 in chain A was also slightly shifted as compared to the crystal structure. In case of chain B, the orientation of PF-07321332 and compound 1 were fit to that of the crystal structure. However, the location of compound 2 was flipped between trifluoromethyl group and tertiary butyl group. The interactions inside the binding pocket of these compounds are shown in Fig. 7.

Due to the modification of cyano group to carbonyl group, compound 1 formed the interaction to the similar surrounding residues as found in PF-07321332. Compound 1 in both chains also showed the interaction to the key residues which are His41 (H-bonds in both chains) and Cys145 (van der Waals in chain A and H-bond in chain B). The modified carbonyl group also revealed the additional H-bond interaction to Gln189 in chain A and to Ser144 and Cys145 in chain B. In case of the compound 2, it exhibited the similar interaction to the residues in the binding site. Considering to the key interaction residues, the H-bond interactions to His41 and Cys145 were found in both chains of the MD structure. However, the H-bond interaction between the modified hydroxyl group and the residues was only found in chain A to His41 and Asn142. In order to confirm for the binding, the binding free energies (∆G_binding_) using MM-PBSA method of all compounds are shown in Table 2. Van der Waals interactions were found to be the key interaction of these compounds. The average binding energies indicated that compound 1 had the stronger binding interaction to the SARS-CoV-2 main protease than PF-07321332 and compound 2, respectively.

**Table 2:** Binding energies and energy components using MM-PBSA method.

Energy terms	Binding energy (kJ/mol)					
	PF-07321332		Compound 1		Compound 2	
	Chain A	Chain B	Chain A	Chain B	Chain A	Chain B
∆E_vdw_	− 157.3 ± 0.3	− 230.4 ± 0.4	− 192.7 ± 0.4	− 229.9 ± 0.4	− 149.1 ± 2.8	− 129.1 ± 2.7
∆E_elect_	− 5.3 ± 0.1	− 21.1 ± 0.1	− 10.9 ± 0.1	− 15.7 ± 0.1	− 10.5 ± 0.3	− 6.0 ± 0.2
∆G_polar_	91.3 ± 0.2	121.3 ± 0.2	104.6 ± 0.3	122.0 ± 0.3	87.0 ± 1.8	67.7 ± 1.2
∆G_nonpol (SASA)_	− 16.5 ± 0.0	− 20.6 ± 0.0	− 20.1 ± 0.0	− 20.5 ± 0.0	− 15.4 ± 0.3	− 13.9 ± 0.3
∆G_binding_	− 87.8 ± 0.4	− 150.8 ± 0.4	− 119.1 ± 0.4	− 144.2 ± 0.4	− 88.1 ± 1.6	− 81.4 ± 2.0
Average	− 119.3		− 131.6		− 84.73	

Where ΔE_vdw_ is the van der Waals interactions, ΔE_elect_ is the electrostatic interactions, ΔG_polar_ is the polar contributions to the solvation free energy, ΔG_binding_ is the binding free energy, ΔG_nonpol_ is the non-polar contributions to the solvation free energy, estimated by solvent accessible surface area (SASA).

## Conclusion

Our focus is to assist in the design of both pan-species and selective antivirals against M^pro^ SARS-CoV-2. The methods are divided into finding the features from frequently occurring atoms that shared across various coronaviral species for the design of pan-variant inhibitors and finding the parts that are specific to either SAR-CoV or SAR-CoV-2 structures. These features may allow for species-specific inhibitor design. Our analysis provides an approach to identify the specificity parts versus the promiscuous parts. This computational approach will allow the dissection of the parts to focus on for other protein targets if there are enough structures, and hence will be useful for future pandemics. As suggested by the superposition on the 4-dimensional grid method, two different types of oxygen containing groups (carbonyl or hydroxyl) were modified to put oxygen at the position of the NH group of PF-07321332 (Paxlovid), i.e. the N5 position from PDB ligand 3-letter code: 4WI. As suggested by the 4D-grid (Fig. 2a) and also Goldscores in this study, the replacement of nitrogen (N5) with an oxygen from carbonyl group resulted in better binding in comparison with either original NH from cyano group of PF-07321332 or hydroxyl (OH) group at N5 of PDB 3-letter code:4WI. The molecular docking for both covalent and non-covalent binding, MD simulations, and binding energies using MM-PBSA methods suggested that having a C=O group replacement could result in better binding based on the frequency of atoms from the mesh (Fig. 2a). All these experiments strongly supported our proposed carbonyl replacement which performed better than paxlovid’s Nirmatrelvir both in terms of binding affinities and capability as a pan-variant inhibitor. Taken together, we proposed that the replacement of NH from cyano group of PF-07321332 of Paxlovid with C=O can result in better binding energy as supported by recent IC_50_ values from Vankadara, et al., for binding experiment with M^pro^ (3CL^pro^) from both SARS-CoV-2 and Human Coronavirus HCoV 229E^45^. The carbonyl oxygens around this location are observed among a variety of input M^pro^ structures (red mesh, Fig. 2a). Interestingly, the inhibitory activity of the proposed compound with the replacement of carbonyl group (compound 1) was confirmed the inhibition against 3CL^pro^ (M^pro^) of SARS-CoV-2 (0.010 µM) and HCoV 229E (0.016 µM)^45^. The experimental inhibitory activity of compound 1 was better than that of Paxlovid, which was agreed well with the binding energies obtained by MM-PBSA method. Our methodology revealed to be the powerful techniques which will be helpful for the development of 3CL^pro^ drugs. Since M^pro^ of Coronavirus has been reported to play a crucial role in viral survival throughout evolution^2,3,9,34^, high conservation of this viral enzyme is hypothesised to be observed at the atomic level as well. The key conserved residues (Pro39, Gly146, and Met162) between SARS-CoV and SARS-CoV-2 M^pro^, which are reported here may be further studied for their position specific parts. Although, the method relies on spatial alignment of X-ray structures, the majority of conserved atoms between this algorithm and our MANORAA algorithm appeared to be in nearby locations (https://manoraa.icbs.mahidol.ac.th/mprocovid/). Data heterogeneity among the structures of M^pro^ in SARS-CoV-2 in various conformations also exists. However, we attempt to use structural ensemble to guide molecular design from the most frequently occurring atoms that are shared across various conformations based on their position specific interactions that are shared among them. By this way, the pan-variant inhibitor can be designed.

The atoms that are conserved in both position and type may not exert direct interaction, but they may be required to control structural conformation and protein stability as they occurred to be position-specific at more than 95% of the M^pro^ SARS-CoV-2 structures (Table 1). It is worth reporting that Cys38 and Val202 have been identified as the highest position-specific amino acids (from location of their nitrogen and alpha carbon atoms at 98.24% conservation). Structure analysis of Val202 and its neighbouring space showed that the most frequently occurring atom of the residue is embedded in the hydrophobic groove, suggesting that Val202 plays an important role in structural stability via the hydrophobic environment. Due to the occurrence of new variants of concern and uncertainty of the severity and symptoms upon infection, the need for novel and precise development of an M^pro^ inhibitor remains. Insights from this study might contribute to the drug discovery community as the specific atomic locations as well as the guides for the ligand design are reported herein. To further demonstrate the application of the identified conserved atoms, SIMFONEE provides a blueprint for the location where all deposited small molecule drugs of SARS-CoV-2 M^pro^ may be bound. Satisfactorily, ligand 4WI of the emergency M^pro^ inhibitor by Pfizer (PF-07321332) clearly occupies the imaginary shape of the ligand in the pocket in Protomer A (between Domain 1 and Domain 2, pocket of S1, S2 and S4)^41^. Structures from the PDB in our dataset covers a wide-range of inhibitors (Supplementary 1), and we have aggregated this position-specific statistical information to increase the signal to noise ratio by counting atoms that are conserved in position and type from M^pro^ structures in the datasets. The possibility of atom replacement is supported by molecular docking and Molecular Dynamics simulation. In order to further validate the identified atomic locations and active site boundary blueprint of SARS-CoV-2 M^pro^, medicinal chemists may further change the molecular interaction of a specific compound by focusing on the guided regions and atoms. The insights from this study in the Supplementary Video 1 might pave the way to new discovery of a more specific inhibitor or ones that are capable of binding across several variants. Our algorithm can be an alternative approach to analyse pan-variant inhibitor to prepare for any future pandemics.

## Supplementary Information

Supplementary Legends.

Supplementary Information 1.

Supplementary Information 2.

Supplementary Video 1.

## Data Availability

The published article includes all data generated during this study in the Supplementary files. All the density functions provided in this manuscript can be accessed on https://manoraa.icbs.mahidol.ac.th/mprocovid/.
