# Supplementary material for: Structural analysis of the coronavirus main protease for the design of pan-variant inhibitors
Source: Sci Rep. 2023 Apr 29;13:7055. doi: 10.1038/s41598-023-34305-6 (PMC10148699; doi:10.1038/s41598-023-34305-6)
Supplement: Supplementary file 1 — Supplementary Legends. [file 41598_2023_34305_MOESM1_ESM.docx]

**Supplementary Materials:**

Supplementary 1: The details of 102 SARS-CoV and 397 SARS-CoV-2 M^pro^ structures obtained from PISCES (.xlsx)

Supplementary 2: The phylogenetic tree generated by iTOL with the heatmap showing the percentage identity of each sequence to SARS-CoV-2 M^pro^, net charge, and molecular mass, as well as the multiple sequence alignment

Supplementary Video 1: Active site analysis of M^pro^ from SARS-CoV and SARS-CoV2 features for unique part for species-specific design and promiscuous parts for pan-coronaviral inhibitor design (.mp4).
