# Supplementary figures and images for: Structural analysis of the coronavirus main protease for the design of pan-variant inhibitors
Source: Sci Rep. 2023 Apr 29;13:7055. doi: 10.1038/s41598-023-34305-6 (PMC10148699; doi:10.1038/s41598-023-34305-6)

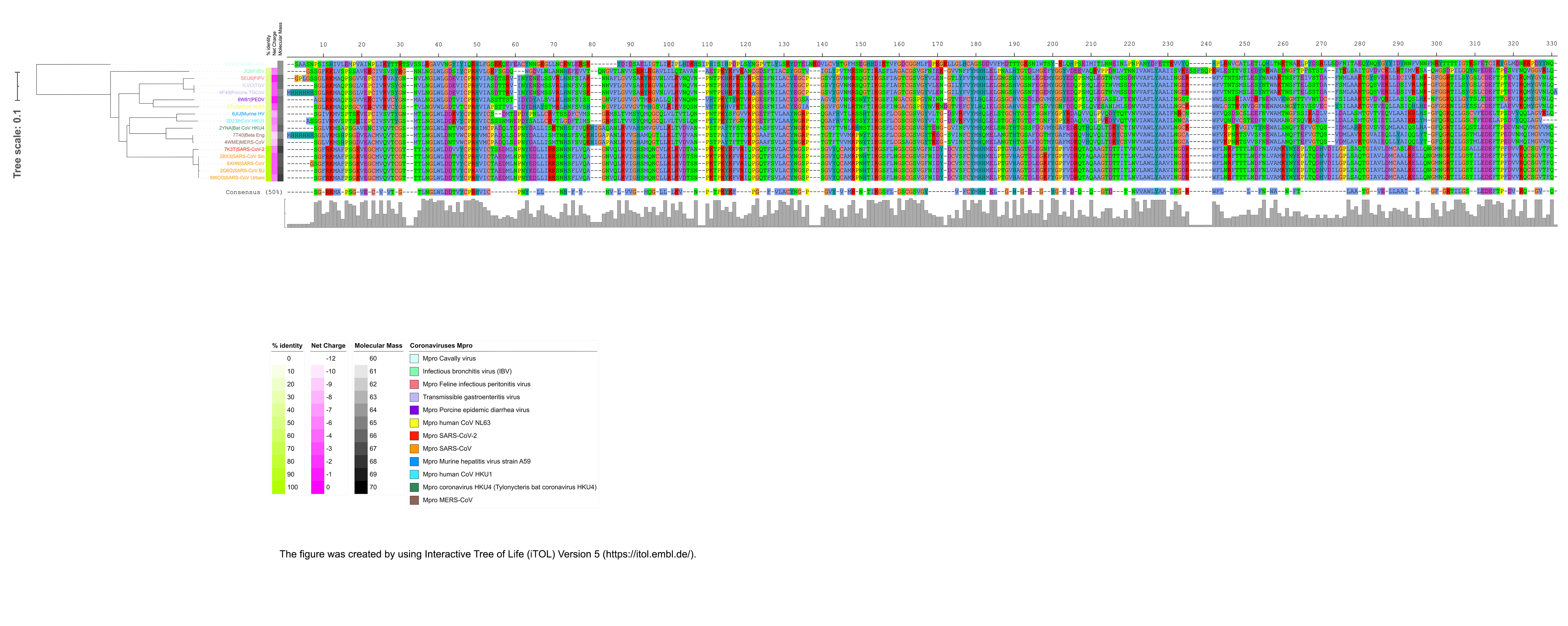

Supplement: Supplementary file 3 — Supplementary Information 2. [file 41598_2023_34305_MOESM3_ESM.jpg]
